# A comprehensive combined experimental and computational framework for pre-clinical wear simulation of total knee replacements

**DOI:** 10.1016/j.jmbbm.2017.11.022

**Published:** 2018-02

**Authors:** A. Abdelgaied, J. Fisher, L.M. Jennings

**Affiliations:** iMBE, University of Leeds, UK

**Keywords:** Wear, Moderately cross-linked ultra-high molecular weight polyethylene, Total knee replacements, Standard kinematics, Deep squat, Stairs ascending

## Abstract

A more robust pre-clinical wear simulation framework is required in order to simulate wider and higher ranges of activities, observed in different patient populations such as younger more active patients. Such a framework will help to understand and address the reported higher failure rates for younger and more active patients (National_Joint_Registry, 2016). The current study has developed and validated a comprehensive combined experimental and computational framework for pre-clinical wear simulation of total knee replacements (TKR).

The input mechanical (elastic modulus and Poisson’s ratio) and wear parameters of the moderately cross-linked ultra-high molecular weight polyethylene (UHMWPE) bearing material were independently measured from experimental studies under realistic test conditions, similar to the loading conditions found in the total knee replacements. The wear predictions from the computational wear simulation were validated against the direct experimental wear measurements for size 3 Sigma curved total knee replacements (DePuy, UK) in an independent experimental wear simulation study under three different daily activities; walking, deep squat, and stairs ascending kinematic conditions.

The measured compressive mechanical properties of the moderately cross-linked UHMWPE material were more than 20% lower than that reported in the literature under tensile test conditions. The pin-on-plate wear coefficient of moderately cross-linked UHMWPE was significantly dependant of the contact stress and the degree of cross-shear at the articulating surfaces.

The computational wear predictions for the TKR from the current framework were consistent and in a good agreement with the independent full TKR experimental wear simulation measurements, with 0.94 coefficient of determination of the framework. In addition, the comprehensive combined experimental and computational framework was able to explain the complex experimental wear trends from the three different daily activities investigated. Therefore, such a framework can be adopted as a pre-clinical simulation approach to optimise different designs, materials, as well as patient’s specific total knee replacements for a range of activities.

## 1 Introduction

The number of younger and more active patients requiring total knee replacements (TKR) is increasing (National_Joint_Registry, 2016). The number of recorded TKR revisions in 2015 in the United Kingdom was 6104 (National_Joint_Registry, 2016). Unsurprisingly, the revision rate for young patients (under 60 years) was 10 times that for patients over 75 years, with more than 20% of the revisions attributed to implant wear (National_Joint_Registry, 2016). More advanced pre-clinical wear simulation methods are therefore needed to assess the wear performance of TKR under a wider range of physiological conditions, simulating the more demanding activities of younger and more active patients.

Pre-clinical pin-on-plate and pin-on-disk testers have been extensively used to screen the performance and explore the influence of parameters such as lubricant, sliding distance, contact stress, and cross-shear ratio on the wear of orthopaedic bearing materials (Barbour et al., 1995, Saikko, 2006, Saikko, 2014, Abdelgaied et al., 2013b, Zhang et al., 2015, Brockett et al., 2016a). Although pre-clinical pin-on-plate and pin-on-disk studies are usually run under simplified test conditions and geometry configurations they provide significant insights into wear characteristics and wear mechanisms of the articulating as well as fixation interfaces of the bearing materials (Zhang et al., 2015, Brockett et al., 2016a). In addition, pre-clinical pin-on-plate and pin-on-disk studies provide the input parameters and validation required for reliable and accurate pre-clinical computational simulation studies (Fregly et al., 2005, Willing and Kim, 2009b, Abdelgaied et al., 2011, Abdelgaied et al., 2013a).

In an attempt to understand and address the higher failure rates reported for young patients, pre-clinical experimental testing methods which include a wider range of physiological conditions have been developed (Jennings et al., 2007, Nakamura et al., 2009, Schwiesau et al., 2013, r et al., 2014). In contrast to the pin-on-plate and pin-on-disk testers, experimental wear tests are run on the full size replacement and under complex and physiologically relevant test conditions (Galvin et al., 2009, Fisher et al., 2010, Brockett et al., 2012, Brockett et al., 2016b, Jennings et al., 2012). Such in-vitro testing is an invaluable method for evaluating bearing materials and total knee replacement geometries. Experimental wear testing has however substantially associated cost and is time consuming, due to the large number of low frequency gait cycles that must be run (Knight et al., 2007).

Computational wear modelling has been extensively used for pre-clinical wear simulation in TKR (Barbour et al., 1995, Fregly et al., 2005, Abdelgaied et al., 2011, Abdelgaied et al., 2014, Brockett et al., 2013, Brockett et al., 2016b), with the low cost and time as well as its appropriateness for parametric studies (Willing and Kim, 2009b, Abdelgaied et al., 2011). Based on wear factor, sliding distance, applied load, contact area, and contact stress, the simplified (Barbour et al., 1995, Maxian et al., 1996, Knight et al., 2007, Pal et al., 2008, Zhao et al., 2008) as well as modified (Willing and Kim, 2009b, Innocenti et al., 2014) versions of Archard’s wear law (Archard and Hirst, 1956) have been adopted in many studies to predict wear in total joint replacements. The applicability of Archard’s wear law to total joint replacements has been questioned (Galvin et al., 2009, Fisher et al., 2010, Liu et al., 2010, Abdelgaied et al., 2011, Innocenti et al., 2014). In addition, the majority of these wear models utilised a wear factor which was chosen from literature to match the experimental measurements. These models are therefore not independent of the experimental simulations, and hence are not validated. Wear factor based computational wear models have therefore shown a limited predictability when running other conditions than the ones they were adapted to simulate (Abdelgaied et al., 2011).

The type of motion at the articulating surfaces in TKR has also been shown to have a significant effect on the wear rate of polyethylene bearings (Wang et al., 1998, Wang, 2001, Kang et al., 2008a, Kang et al., 2008b, Abdelgaied et al., 2013b). The cross-shear parameter was developed to describe the significant effect the multidirectional motion had on polyethylene wear, compared to unidirectional motion (Bragdon et al., 1996, Wang, 2001). The reported wear parameters under multidirectional motions were up to ten times more than that under unidirectional motion, depending on the degree of cross-shear at the articulating surfaces (Wang, 2001, Kang et al., 2008b, Abdelgaied et al., 2013b). However, the simplified Archard’s wear law, and therefore the simplified Archard’s wear law based models, does not account for these cross-shear effects.

The input mechanical properties of the total knee replacement bearing materials, such as elastic modulus and Poisson’s ratio, significantly contribute to the predictability of computational models. They should ideally be determined from independent experimental studies, under similar test conditions to the clinical and experimental conditions, to provide reliability and validity to the computational models. In most cases, the reported values in the literature for the elastic modulus and Poisson’s ratio of the bearing materials have been measured under tensile test conditions, in contrast to the compressive operating conditions of the total knee replacements (Bei et al., 2004, Bevill et al., 2005, Fregly et al., 2005, Jourdan, 2006, Zhao et al., 2006, Zhao et al., 2008, DE Jongh et al., 2008, Pal et al., 2008, Carr and Goswami, 2009, Jourdan and Samida, 2009, Kang et al., 2009, Willing and Kim, 2009a, Willing and Kim, 2009b, Innocenti et al., 2014).

In addition, clinical, experimental, and computational studies have reported increased polyethylene wear rate under high contact stress conditions (Griffin et al., 1998, Foran et al., 2004, Amin et al., 2006, Kang et al., 2008a, Kang et al., 2009, O’brien et al., 2015). In most cases, the input wear parameters to the computational models have been experimentally measured under average contact stresses to simulate standard activities. These wear studies are not therefore applicable for more adverse conditions, including higher levels of activities and severe loading conditions.

The aim of the current study was to develop and experimentally validate a new fully independent framework for pre-clinical wear simulation in total knee replacements. The input mechanical and wear parameters of the bearing materials were determined from independent experimental studies of material and wear properties under wider and more realistic test conditions. Our hypothesis was that the new fully independent framework would be a more reliable computational prediction of the wear of the polyethylene in TKR and provide a better agreement with the full TKR experimental simulation measurements of TKR wear.

## 2 Materials and methods

**Fig. 1:**
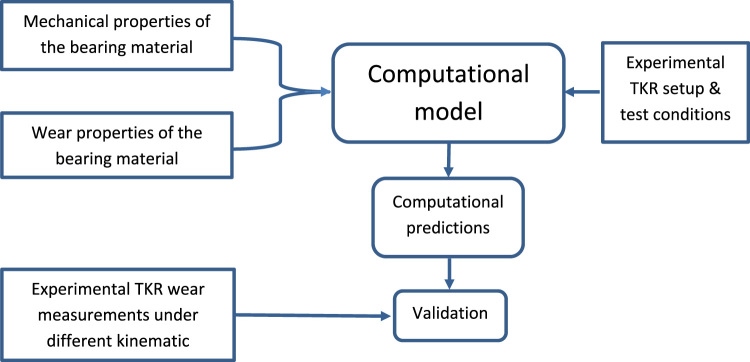
Combined experimental and computational framework for pre-clinical simulation of total knee replacements.

This study developed combined experimental and computational simulation methods to develop and validate a fully independent framework for pre-clinical wear simulation in TKR. In this approach, the experimental mechanical, pin-on-plate, and knee simulation studies provided the inputs as well as the validation to the computational wear model. The pre-clinical wear simulation framework proposed in the current study is summarised in Fig. 1.

### 2.1 Wear model

Based on the modification of Archard’s law where wear volume (W) is proportional to the contact area (A) and sliding distance (S) (Liu et al., 2010), the wear volume was defined as:

(1) W=A×S×C

where C is a non-dimensional wear coefficient.

Clinical and experimental wear studies have shown that wear is dependant on the cross-shear ratio (CS) and the contact stress (P) at the articulating surfaces (Wang, 2001, Foran et al., 2004, Kang et al., 2008b, O’brien et al., 2015). The non-dimensional wear coefficient was therefore defined as a function of CS and a non-dimensional contact stress (P/E), where E is the elastic modulus of the polyethylene baring material:

(2) $$C=\text{fun}(\text{CS},\frac{P}{E})$$

The linear wear depth (δ) can also be derived from Eq. (1) as:

(3) $$\delta =S\times \text{C}\;(\text{fun}(\text{CS},\;\frac{P}{E}))\;$$

Based on the unified theory of wear and frictional work by Wang (2001), the cross-shear ratio was defined as the frictional work component perpendicular to the principal molecular orientation (PMO) direction (E_cross-shear_), divided by the total frictional work (E_total_), thus:

(4) $$\mathrm{CS}=\frac{E_{\mathrm{cross}-\mathrm{shear}}}{E_{\mathrm{total}}}$$

The non-dimensional wear coefficient (C), a function of CS and non-dimensional contact stress (P/E), was measured from an independent experimental pin-on-plate wear study of the same material combination as the total knee replacement articulating bearing materials (Section 2.2).

Eq. (3) was used to predict the wear of moderately cross-linked ultra-high molecular weight polyethylene (UHMWPE) inserts (GUR 1020, 5 Mrad gamma irradiation). Size 3 DePuy Sigma fixed bearing TKRs (DePuy, UK) were used, with 10 mm thick curved inserts. The moderately cross-linked UHMWPE material was modelled as an elastic material, using the equivalent elastic modulus and Poisson’s ratio from an independent mechanical properties study (Section 2.3). The Cobalt-Chrome (CoCr) femoral component was modelled as a rigid body.

A finite element simulation model was developed in ABAQUS (ABAQUS, v6.14-1, USA) to simulate the experimental test conditions. The tibial and the femoral components were meshed using quadratic tetrahedral elements (C3D10M). The mesh sensitivity study resulted in total number of elements of 161,367 and 23,438 for the tibial and the femoral components respectively. An isotropic coefficient of friction of μ = 0.04 was assumed (Godest et al., 2002, Willing and Kim, 2009b) in a penalty contact formulation to describe the contact between the tibial and femoral contact surfaces. The gait cycle of 1 s was divided into 128 steps. The load was applied on a control node of the femoral component. The control node was determined at the axis through the centre of the femoral component and offset by 7% of its width in the medial direction, in accordance with the experimental setup and the ISO recommendation (ISO-14243-1, 2009, ISO-14243-3, 2014). The flexion-extension was also prescribed through the control node of the femoral component. All other degrees of freedom of the femoral component were constrained. The tibial and the femoral contact surfaces were brought into contact at their lowest points. Anterior-posterior displacement as well as the internal-external rotation was applied to the UHMWPE insert control node. The control node was determined in accordance with the experimental setup. Adduction–abduction motion was allowed and unconstrained. All other degrees of freedom of the tibial component were constrained.

The predictions from the finite element contact analysis in ABAQUS, including the sliding distance and contact stress at each node and each time increment as well as the contact area, were used to estimate the linear wear depth at each node as well as the volumetric wear at each element on the moderately cross-linked UHMWPE insert surface, using an in house developed Matlab code. A Matlab script was also developed to interact with the ABAQUS input file and update the UHMWPE insert surface nodes to its new positions as the wear propagated. The UHMWPE insert surface was updated every 500,000 cycles, using the total linear wear at each node. The computational simulations were run to the same number of cycles as the full TKR experimental simulations and the average volumetric wear rates were calculated and compared to the full TKR experimental measurements.

### 2.2 Pin-on-plate material wear study to determine input parameters

An independent experimental pin-on-plate wear study, of the same material combination as the total knee replacement articulating bearing materials, has been conducted. This pin-on-plate study investigated the multidirectional wear performance of moderately cross-linked UHMWPE under a wide range of applied nominal contact stresses and different levels of cross-shear at the articulating surfaces. The measured non-dimensional wear coefficient of the moderately cross-linked UHMWPE as a function of CS and a non-dimensional contact stress was an input to the computational wear model.

**Fig. 2:**
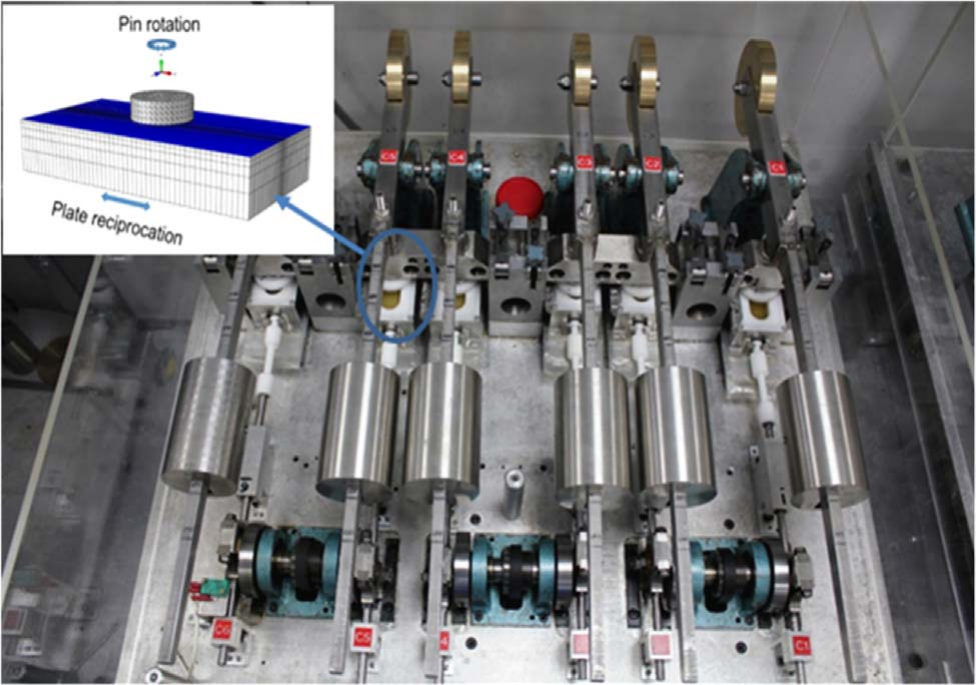
Multidirectional pin-on-plate wear simulator.

Moderately cross-linked UHMWPE (GUR 1020, 5 Mrad gamma irradiation) cylindrical pins were tested against CoCr plates in a multidirectional pin-on-plate wear simulator (Fig. 2). The CoCr metallic plates were polished to an average surface roughness Ra of 0.01 µm. Six different pin diameter and applied load combinations were tested, resulting in applied nominal contact stresses ranging from 4 to 80 [MPa]. The pin rotation and the plate reciprocation were in phase, having a common frequency of 1 Hz, and resulted in a multidirectional motion at the pin-plate contact surface in a flat-on-flat configuration. Five different pin rotation and plate reciprocation combinations were tested, resulting in five different degrees of cross-shear at the articulating surfaces, ranging from 0 (unidirectional motion) to 0.18. The pin-on-plate test conditions are summarised in Table 1.

**Table 1:** Pin-on-plate test conditions.

Pin diameter [mm]	Load [N]	Stress [MPa]	Non-dimensional stress (P/E) [--]	Stroke length [mm]	Rotation angle [degrees]	CS	Test period [weeks]
5	80	4	0.007	28	±30	0.087	3
5	216	11	0.020	28	±30	0.087	3
4	252	20	0.036	28	±30	0.087	3
3	212	30	0.054	28	±30	0.087	3
3	283	40	0.071	28	±30	0.087	3
2	252	80	0.143	28	±30	0.087	3
5	216	11	0.020	28	0	0.0	2
5	216	11	0.020	10	±10	0.01	2
5	216	11	0.020	12	±15	0.022	2
5	216	11	0.020	26	±45	0.18	2
5	80	4	0.007	10	±10	0.01	2
2	252	80	0.143	10	±10	0.01	2
4	252	20	0.036	12	±15	0.022	2
4	252	20	0.054	12	±15	0.022	2

Six pins were tested for each condition and each condition was run for at least two weeks (660,000 cycles) in 25% bovine serum as a lubricant. The volumetric wear was calculated from the weight loss measurements using a density of 0.93 mg/mm^3^ for the UHMWPE material. The wear coefficient was calculated using Eq. (1) as:

(5) $$\text{C}\;(\text{fun}(\text{CS},\;\frac{P}{E}))=\frac{W}{A\times S}$$

Statistical analysis of the data was performed in ANOVA and significance was taken at p < 0.05. In addition, the measured wear coefficient was expressed as a function of the CS and non-dimensional contact stress (P/E) using OriginLab program (OriginPro 8.5.1).

### 2.3 Mechanical properties of the polyethylene bearing material to determine input parameters

**Fig. 3:**
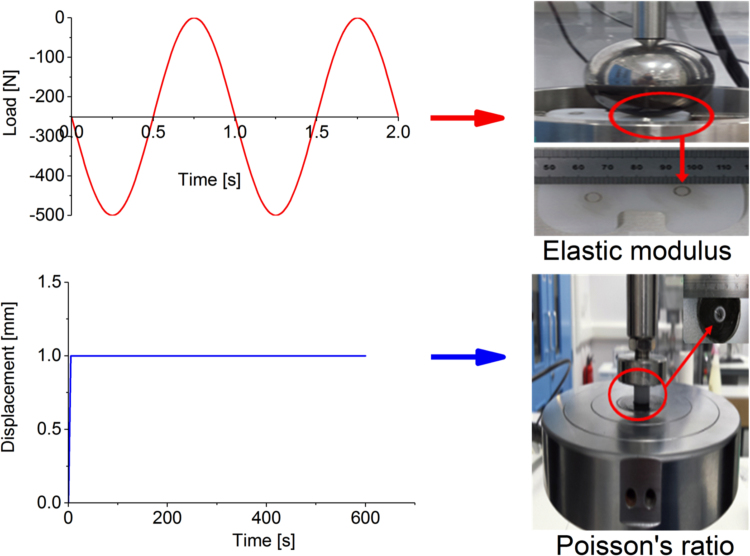
Poisson’s ratio and equivalent elastic modulus experimental tests.

An independent combined experimental and computational approach was developed to measure mechanical properties of the moderately cross-linked UHMWPE articulating surface under realistic compressive test conditions, similar to the operating condition of total joint replacements. To determine the Poisson’s ratio of the moderately cross-linked UHMWPE, contact areas of 12 mm diameter cylindrical specimens of 10.2 mm length, resting against infinitely rigid flat steel cylinders, were measured experimentally under a compressive displacement of 1 mm, to achieve similar contact stress (~35 MPa) to that in total knee replacements (Fig. 3) (Godest et al., 2002, Galvin et al., 2009). The compressive displacement was applied at a strain rate of 12 mm/min and was held for 10 min, using an E10000 electropuls Instron (Instron, UK). Compressive displacement was used, instead of compressive force, so that the measured contact area was only dependant on the Poisson’s ratio and independent of the elastic modulus of the moderately cross-linked UHMWPE cylindrical specimens. A computational model was developed in Abaqus to simulate this experimental test assuming different values for the Poisson’s ratio of the UHMWPE cylindrical specimens. The curve fit relationship between the computationally predicted contact area and Poisson’s ratio was used to calculate the Poisson’s ratio of the UHMWPE specimens, using the experimentally measured contact areas (Image Pro, v6.3, USA).

Using a similar approach, the equivalent elastic modulus of the UHMWPE was calculated using the computationally calculated curve fit contact area-elastic modulus relationship, from the computational simulation of a ball-on-flat compression test, and the experimentally measured contact area from a ball-on-flat dynamic compression test. This experiment used 10 mm thick moderately cross-linked UHMWPE flat specimens against a 63.5 mm infinitely rigid steel ball, under a compressive dynamic sinusoidal loading of 250 N average load (from zero to 500 [N] load), and 6000 cycles, using an E10000 electropuls Instron (Instron, UK). The applied test conditions maintained the stress level within the reported range for TKR (~30 – 40 MPa) (Godest et al., 2002, Galvin et al., 2009).

### 2.4 Experimental full TKR wear simulation for validation of the computational wear predictions

In order to independently validate the computational wear model, six Sigma fixed bearing cruciate retaining total knee replacements (DePuy, UK) comprising Co-Cr-Mo alloy femoral components, and polished Co-Cr-Mo tibial trays, were used with curved polyethylene tibial inserts. The inserts were moderately cross-linked UHMWPE (XLK™) (GUR 1020, 5 Mrad gamma irradiation). The six sets of bearings were mounted anatomically in each station. The central axis of each implant was offset from the aligned axes of applied load and tibial rotation from the centre of the joint by 7% of its width, in accordance with the ISO recommendation (ISO-14243-1, 2009, ISO-14243-3, 2014). The centre of rotation of the femoral components was taken as the distal radius of the implant, as indicated by the device design.

**Fig. 4:**
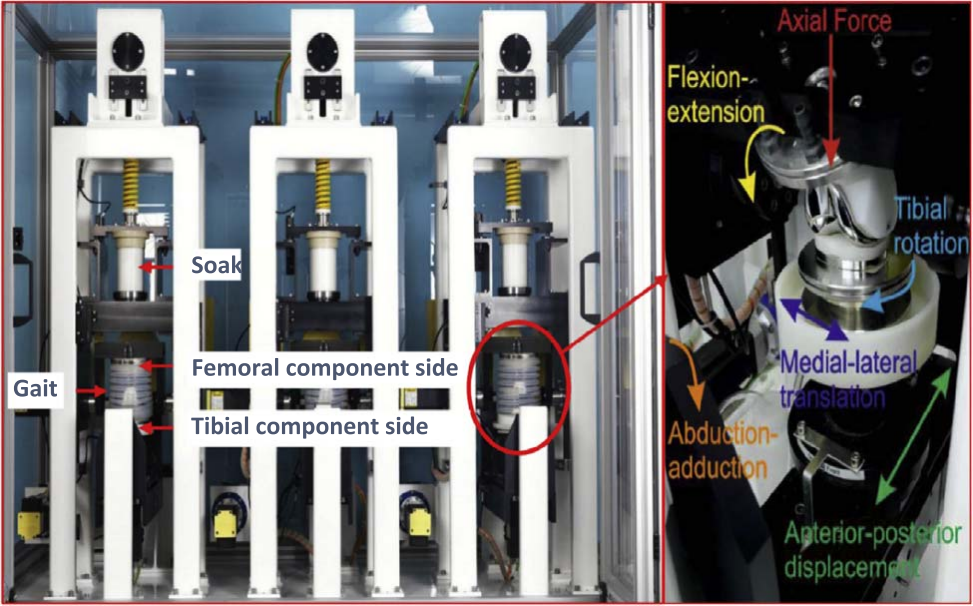
Six station electromechanically driven knee simulator (Simulation Solutions, UK), and the controlled axes of articulation for each station.

The experimental simulation was run using a six station electromechanically driven knee simulator (Simulation Solutions, UK). The simulator had six fully independent stations in two banks; three stations per each bank (Fig. 4). Each station had six degrees of freedom with five controlled axes of motion – axial load to the femoral component, femoral flexion extension, tibial internal/external rotation, tibial anterior-posterior displacement, and tibial adduction-abduction rotation (Abdelgaied et al., 2017a).

**Fig. 5:**
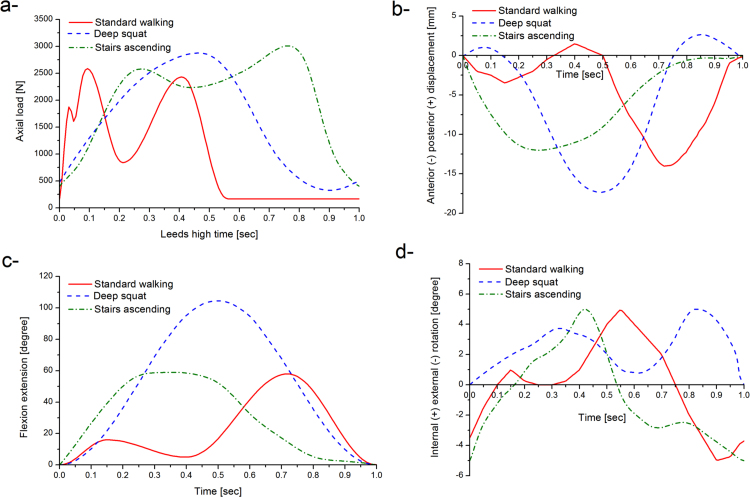
Axial load (a), anterior posterior displacement (b), flexion extension angle (c), and internal external rotation (d) input profiles for standard walking, deep squat, and stairs ascending kinematics.

Three different daily activities were explored through the study, namely standard walking kinematics (Lafortune et al., 1992, Mcewen et al., 2005), deep squat kinematics (Schwiesau et al., 2013), and stairs ascending kinematics (Battaglia et al., 2014), shown in Fig. 5. The maximum axial loads were 2600, 2879, and 3008 [N] for standard walking, deep squat, and stairs ascending kinematics respectively. The maximum anterior posterior translations were 14, 17, and 12 [mm] for standard walking, deep squat, and stairs ascending kinematics respectively. Anterior-posterior translation was displacement controlled, as this design of fixed bearing knee replacement had minimal constraint and thus relies on soft tissue in-vivo. The flexion extension ranges were 0˚ to 58˚, 0˚ to 104˚, and 0˚ to 60˚ for standard walking, deep squat, and stairs ascending kinematics respectively. The corresponding internal external rotation ranges were −5˚ to 5˚, 0˚ to 5˚, and −5˚ to 5˚ respectively (Fig. 4). Internal external tibial rotation was displacement controlled. The femoral distal radius was taken as the femoral centre of rotation with a polarity of anterior tibial shift (denoted as negative anterior posterior motion) that produced femoral rollback. Abduction adduction was allowed but not controlled (Abdelgaied et al., 2017a).

The experimental simulation study was run sequentially for 3 million cycles (mc) of standard walking, 1 mc of deep squat, and 1 mc of stairs ascending kinematics, as the deep squat and the stairs ascending kinematics are less frequent activities compared to the standard walking activity. The simulator was run at a frequency of 1 Hz. The lubricant used was new-born calf serum, diluted to 25%, supplemented with 0.03% (v/v) sodium azide to retard bacterial growth, and was changed every 0.33 mc. Prior to testing, all inserts were soaked in deionised water for a minimum period of four weeks. This allowed an equilibrated fluid absorption level to be achieved prior to the commencement of the wear study, reducing variability due to fluid weight gain. Wear was determined gravimetrically at one million cycle measurement intervals throughout the study. A Mettler XP205 (Mettler-Toledo, USA) digital microbalance, which had a readability of 0.01 mg, was used for weighing the bearing inserts. The volumetric wear was calculated from the weight loss measurements, using a density of 0.93 mg/mm^3^ for the polyethylene material, and using unloaded soak controls to compensate for moisture uptake. The cumulative volumetric wear was calculated for each station and the mean wear rate was then calculated for all 6 stations (mean ±95% Confidence Intervals), of each kinematics. Statistical analysis of the data was performed in ANOVA and significance was taken at p<0.05.

## 3 Results

### 3.1 Mechanical properties of the polyethylene bearing material

**Fig. 6:**
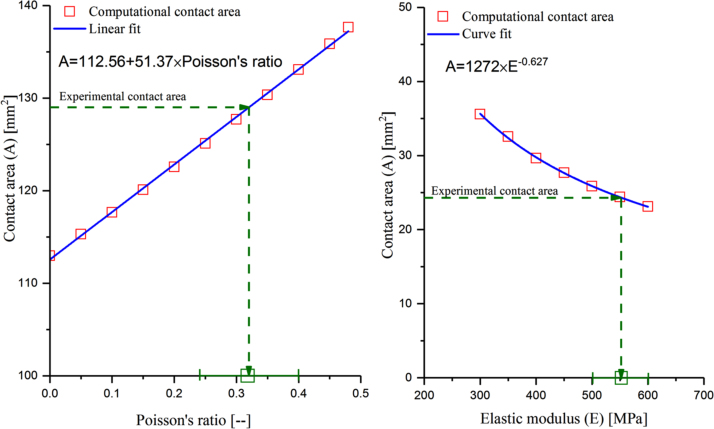
Poisson’s ratio and average elastic modulus for moderately cross-linked UHMWPE under compressive test conditions.

The predicted Poisson’s ratio and equivalent elastic modulus for moderately cross-linked UHMWPE are shown in Fig. 6. The predicted maximum contact stress, under the applied test conditions, was 35 MPa. The predicted Poisson’s ratio was 0.32 ± 0.08 (mean ± 95% CI, n = 5). The predicted equivalent elastic modulus was 553 ± 51 MPa (mean ± 95% CI, n = 5).

### 3.2 Pin-on-plate material wear study

**Fig. 7:**
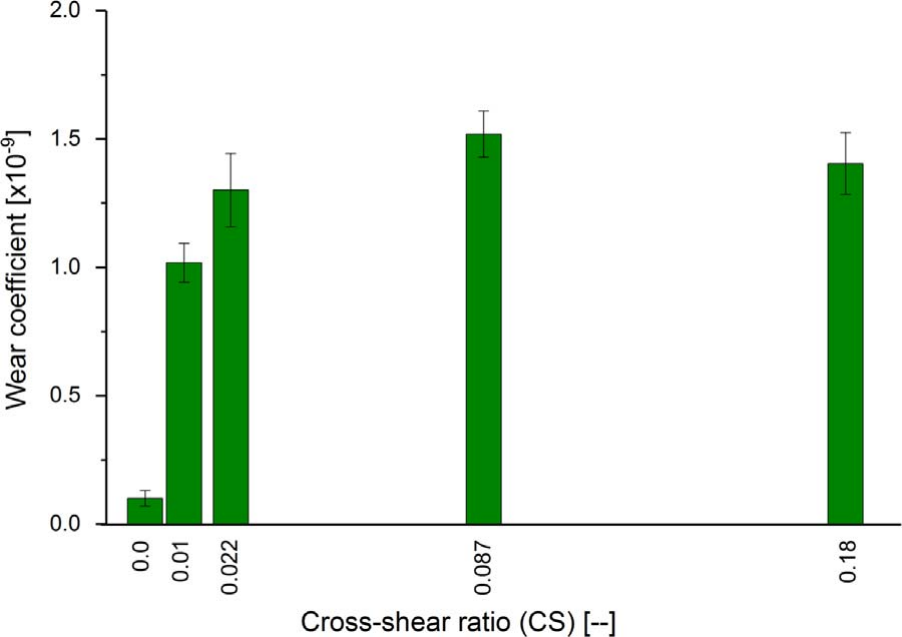
Wear coefficients for moderately cross-linked UHMWPE under different cross-shear ratios (mean ± 95% CI, n = 6), and 11 MPa contact stress.

**Fig. 8:**
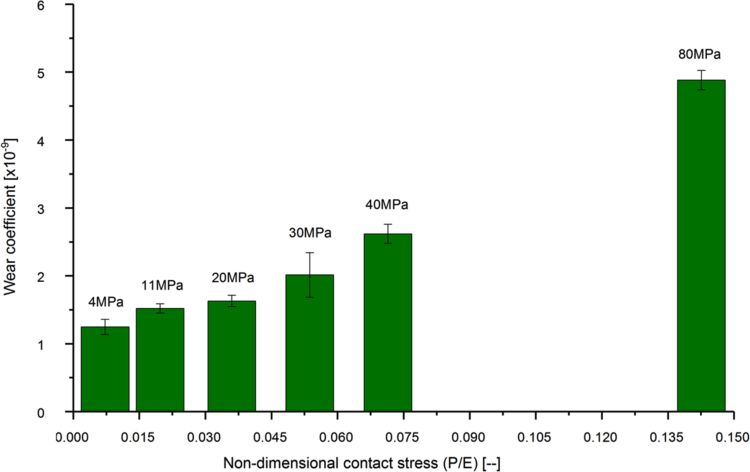
Wear coefficients for moderately cross-linked UHMWPE under different applied non-dimensional contact stresses (mean ± 95% CI, n = 6), and 0.087 cross-shear ratio.

Moderately cross-linked UHMWPE pins were tested in a multidirectional pin-on-plate wear simulator against CoCr plates to provide the input wear coefficient to the computational wear model. Under the same applied nominal contact stress of 11 MPa, changing the CS from zero (28 mm plate reciprocation, zero degree pin rotation) to 0.01 (10 mm plate reciprocation, ±10 degrees pin rotation) significantly increased the wear coefficient from 0.1 ± 0.03 to 1.02 ± 0.08 [x10^-9^] (mean ± 95% CI, n = 6, ANOVA, p < 0.001). Further increase in the CS to 0.18 (26 mm plate reciprocation, ±45 degrees pin rotation) significantly increased the wear coefficient to 1.40 ± 0.12 [x10^-9^] (mean ± 95% CI, n = 6, ANOVA, p < 0.001) (Fig. 7). For the same degree of CS at the articulating surfaces (CS = 0.087), the measured material wear coefficient significantly increased from 1.25 ± 0.11 to 4.88 ± 0.14 [x10^-9^] (mean ± 95% CI, n = 6, ANOVA, p < 0.001) while increasing the non-dimensional stress from 0.007 to 0.02, corresponding to stress values from 4 to 80 [MPa] (Fig. 8).

**Fig. 9:**
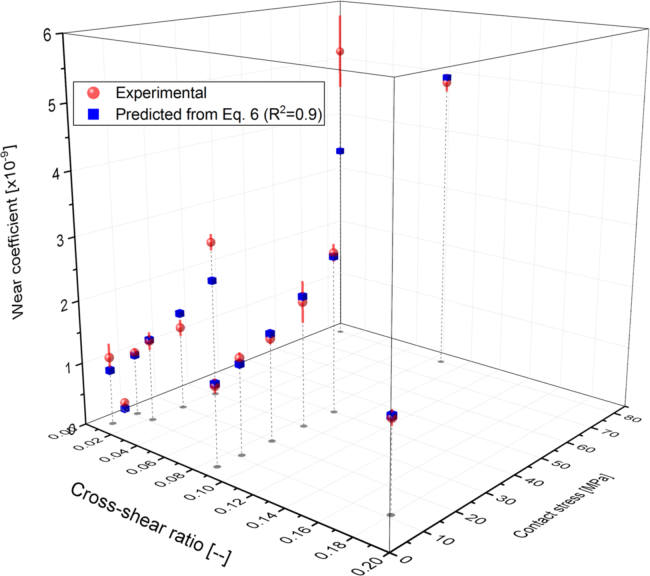
Experimental (mean ± 95% CI, n = 6) and predicted (Eq. (6)) wear coefficients for moderately cross-linked UHMWPE under different cross-shear ratios and different contact stresses.

The non-dimensional wear coefficient as a function of CS and non-dimensional stress was obtained from the 3D best fit of the experimental results using OriginLb (OriginPro 8.5.1). The function expressed by the following equation (Eq. (6)), had the best R-squared value of 0.96 as shown in Fig. 9:

(6) $$C=10^{-9}\times \left[1.47\times \left(1-\exp\left(-116.21\times \mathrm{CS}\right)\right)\times \left(0.84+\mathrm{45}\mathrm{0.23}\times \left({\left(\frac{P}{E}\right)}^{1.49}\right)\right)\right]$$

### 3.3 Computational TKR wear predictions

**Fig. 10:**
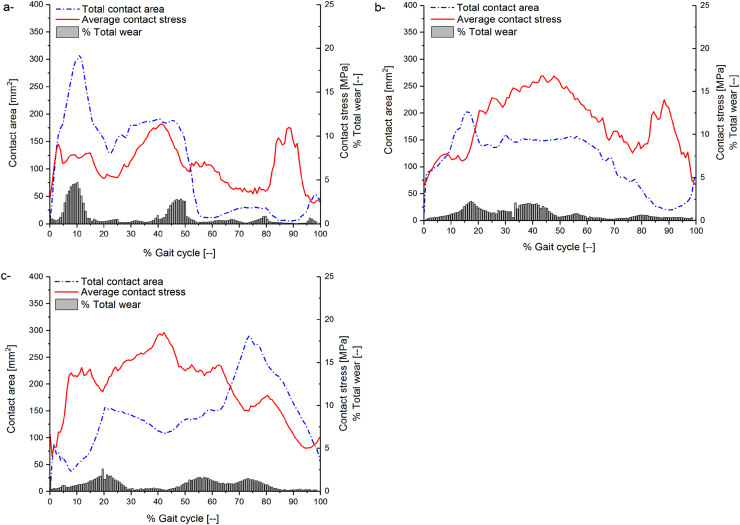
Predicted contact area [mm^2^], average contact stress [MPa], and percentage contribution to the total wear at different percentage of the loading cycle under standard (a), deep squat (b), and stairs ascending (c) kinematic conditions.

The fully independently measured mechanical and wear parameters (3.1, 3.2) were then used in the computational wear model to calculate linear and volumetric wear rates. The predicted volumetric wear rates under standard walking, deep squat, and stairs ascending kinematic conditions are summarised in Table 2. The predicted contact area, average contact stress at different percentage of the loading cycle as well as percentage contribution to the total wear under different test conditions are shown in Fig. 10.

**Table 2:** The predicted volumetric wear rates, average contact area, average contact stress, and average CS under standard walking, deep squat, and stairs ascending kinematic conditions.

Test conditions	Standard walking	Deep squat	Stairs ascending
Volumetric wear rate [mm^3^/mc]	4.5	3.7	5.6
Average contact area [mm^2^]	106	114	145
Average contact stress [MPa]	6.7	11.4	12.2
Average CS [--]	0.06	0.03	0.05

### 3.4 Experimental TKR wear simulation

**Fig. 11:**
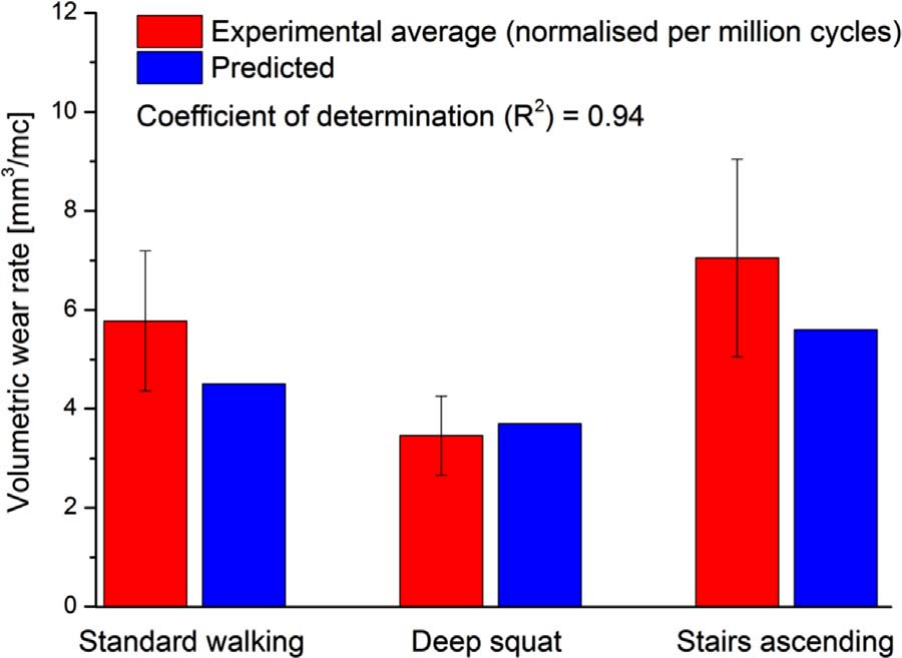
Experimental and computational volumetric wear rates [mm^3^/mc] (mean ± 95% CI, n = 6) under standard walking, deep squat, and stairs ascending kinematic conditions.

The measured average experimental wear rate (normalised per million cycles) under standard walking and stairs ascending kinematics were not significantly different at 5.8 ± 1.4 and 7.1 ± 2.0 [mm^3^/mc] respectively (mean ± 95% CI, n = 6, ANOVA, p > 0.05). The measured average wear rate (normalised per million cycles) under deep squat kinematics was significantly lower, compared to that under standard walking and stairs ascending kinematics, at 3.5 ± 0.8 mm^3^/mc (mean ± 95% CI, n = 6, ANOVA, p < 0.05). The measured experimental wear rates are compared to the computational wear predictions in Fig. 11.

## 4 Discussion

Computational wear modelling has been increasingly and extensively used for pre-clinical wear simulation as well as optimisation of TKR designs, with its appropriateness for parametric studies at low cost and time (Carr and Goswami, 2009, Willing and Kim, 2009b, Abdelgaied et al., 2011). Utilising arbitrarily chosen wear factors, with limited predictability, many studies have adopted simplified as well as modified versions of Archard’s wear law (Archard and Hirst, 1956) to predict wear in TKR. Moreover, the simplified Archard’s wear law, and therefore the simplified Archard’s wear law based models, do not account for the effect of cross-shear on wear of polyethylene. In addition, all the reported wear studies have used mechanical properties for the articulating bearing materials that have been measured under tensile test conditions, as opposed to the compressive operating conditions of TKR. Also, the input wear parameters to the models have been assumed or measured under average contact stresses to simulate standard activities. These models are therefore not independent, have limited predictability when running other conditions than the one they were adapted to simulate, not validated, and are not applicable for more adverse conditions that could lead to edge loading and high stress conditions, including higher levels of activities and severe loading conditions. The current study developed and experimentally validated a new fully independent framework for the pre-clinical wear simulation in total knee replacements. The input mechanical and wear parameters to the computational model were determined from independent experimental studies under wider and more realistic test conditions.

The reported values for Poisson’s ratio and elastic modulus of moderately cross-linked UHMWPE material, measured under tensile test conditions, ranged between 0.4 to 0.46 and 600 to 1000 [MPa] respectively (Bartel et al., 1995, Godest et al., 2000, Godest et al., 2002, Carr and Goswami, 2009, Abdelgaied et al., 2014). The measured Poisson’s ratio and elastic modulus for moderately cross-linked UHMWPE material from the current study, under more realistic compressive test conditions, were more than 20% lower than values reported in literature that have been measured under tensile test conditions (Fig. 6). In addition to the realistic compressive test conditions, the experimental contact area, used in Fig. 6 to predict the equivalent elastic modulus, was measured under dynamic sinusoidal compressive loading conditions and the measured equivalent elastic modulus therefore accounted for the creep of the polyethylene bearing material.

The wear parameters of the bearing materials were measured from an independent multidirectional pin-on-plate wear study. For the same level of motion at the articulating surfaces, the two main parameters that significantly contributed to the volumetric wear were the applied load and contact area. The measured wear parameters were significantly dependant on the applied nominal contact stress and the degree of cross-shear at the articulating surfaces. Under the same applied nominal contact stress of 11 MPa, the measured wear coefficients under multidirectional motions (CS ≠ 0) were more than ten times that under unidirectional motion, depending on the degree of cross-shear at the contact surfaces (Fig. 7). This finding is consistent with other studies emphasised the significant effect of CS on wear of UHMWPE materials and may explain the lower wear prediction of computational wear models, based on wear parameters from unidirectional wear testers (Wang, 2001, Kang et al., 2008b, Abdelgaied et al., 2013b, Brockett et al., 2016a). In contrast to other pin-on-plate wear studies in literature (Kang et al., 2008b, Abdelgaied et al., 2013b, Brockett et al., 2016a), under the same degree of cross-shear at the articulating surfaces, the measured wear coefficient was found to be highly pressure dependant. The measured wear coefficients under a high contact stress of 80 MPa were more than four times higher than their coressponding corresponding values under 4 MPa stress while maintaining the cross-shear at either 0.01 or 0.087 (Fig. 8). These studies, however, were conducted under a limited range of contact stresses of up to 11 MPa maximum contact stress (Kang et al., 2008b, Abdelgaied et al., 2013b, Brockett et al., 2016a). In addition, clinical, experimental, and computational studies reported increased polyethylene wear under high contact stress conditions (Griffin et al., 1998, Foran et al., 2004, Amin et al., 2006, Kang et al., 2009, O’brien et al., 2015).

The independently measured material mechanical and wear parameters of the polyethylene articulating surface were adopted in the computational wear model. The model was used to predict the volumetric wear under three different standard and high levels of activity. The computational model was set to simulate the running and boundary conditions of the independently conducted experimental wear simulation study. The computationally predicted and experimentally measured wear rates were in a good agreement, with 0.94 coefficient of determination of the computational model (Fig. 11). In addition, the measured complex experimental wear trends could be computationally explained by the differences in cross-shear, contact area, and contact stress distributions between different kinematics throughout the loading cycle (Fig. 10). For example, the stairs ascending loading condition had similar (axial load, flexion-extension, and tibial rotation) or lower (anterior-posterior direction translation) level of kinematics, compared to the standard kinematics. In addition, the predicted average cross-shear ratio under stairs ascending kinematics (average CS = 0.05) was lower than that under standard kinematics (average CS = 0.06). However, the stairs ascending kinematics produced a higher wear rate compared to the standard kinematics (the results were not however significantly different (ANOVA, p > 0.05)). This could be explained by the increased contact stresses predicted throughout the loading cycle (Fig. 10). Similarly, the deep squat kinematics had higher axial load, flexion-extension, and anterior-posterior kinematics, but lower tibial rotation, compared to standard and stairs ascending kinematics. The deep squat however had a significantly lower wear rate compared to that of standard and stairs ascending kinematics (ANOVA, p < 0.05). This could be explained by the low average cross-shear ratio predicted under deep squat kinematics (average CS = 0.03) compared to that predicted under standard and stairs ascending kinematics (average CS = 0.06). The computational wear predictions from the current framework are compared to the computational wear predictions from Brockett et al. (2016b) in Table 3.

**Table 3:** The predicted volumetric wear rates, average contact area, contact stress, and CS under standard walking, deep squat, and stairs ascending kinematic conditions.

Test conditions	Standard walking	Deep squat	Stairs ascending
Computational wear prediction from the current model [mm^3^/mc]	4.5	3.7	5.6
Computational wear prediction from Brockett et al. (2016b)	3.4	2.6	4.2
Experimental wear rate	5.8±1.4	3.5±0.8	7.1±2.0

The experimental pin-on-plate wear study was conducted under static loading conditions, in contrast to the dynamic experimental/clinical knee loading conditions. In addition, the UHMWPE pin-on-plate wear study was conducted against very smooth CoCr plates (average surface roughness of 0.01 µm), which is difficult to obtain on cast CoCr femoral surfaces (Liu et al., 2010, Abdelgaied et al., 2011). Dynamic loading conditions and rougher plates in a pin-on-plate wear test could increase the measured wear parameters and therefore the computational wear predictions (Barbour et al., 1997, Liu et al., 2010, Abdelgaied et al., 2011). Future work will address the effect of surface roughness of the CoCr plates and the nature of the loading conditions on measured wear parameters from pin-on-plate wear testers.

Most importantly, the current study presented a comprehensive combined experimental and computational framework for pre-clinical wear simulation in total knee replacements, validated for three different daily activities. The framework was also able to predict the change in surface wear associated with change in kinematic conditions. The predicted wear rates were consistent and in a good agreement with the experimental measurements (the coefficient of determination of the framework = 0.94). However, the changes found in the model, did not fully predict the changes found experimentally, indicating other factors in the experimental model, not yet incorporated in the model, such as plastic deformation of polyethylene material, may play an additional role experimentally in high demand activities.

Overall, the new comprehensive combined experimental and computational framework has been shown to be a robust pre-clinical wear simulation approach capable of predicting as well as explaining complex experimental wear trends. It should be emphasised again that the new combined experimental and computational framework is independent and the mechanical properties as well as the wear parameters of the articulating bearing materials were taken from independent mechanical and pin-on-plate wear studies. Therefore, such a framework can be adopted and applied as a pre-clinical simulation approach to optimise different designs, materials, as well as patient specific total knee replacements.

## 5 Conclusion

The current study developed and validated a comprehensive combined experimental and computational pre-clinical wear simulation framework for total knee replacements. The mechanical and wear parameters of the bearing materials were measured from independent combined experimental and computational studies under wider and more realistic test conditions. The wear predictions were validated against the measurements from an independent experimental simulation study under three different daily activities, giving a coefficient of determination of 0.94. Future work will apply the developed framework to pre-clinically optimise different designs, materials, as well as patient specific total knee replacements.
